# Full-Field Strain Mapping at a Ge/Si Heterostructure Interface

**DOI:** 10.3390/ma6062130

**Published:** 2013-05-24

**Authors:** Jijun Li, Chunwang Zhao, Yongming Xing, Shaojian Su, Buwen Cheng

**Affiliations:** 1 College of Science, Inner Mongolia University of Technology, Hohhot 010051, China; E-Mails: lijjtom@yahoo.com.cn (J.L.); xym@imut.edu.cn (Y.X.); 2 College of Information Science and Engineering, Huaqiao University, Xiamen 361021, China; E-Mail: sushaojian@hqu.edu.cn; 3 State Key Laboratory on Integrated Optoelectronics, Institute of Semiconductors, Chinese Academy of Sciences, Beijing 100083, China; E-Mail: cbw@red.semi.ac.cn

**Keywords:** misfit dislocation, strain, Ge/Si heterostructure, geometric phase analysis, peak pairs analysis

## Abstract

The misfit dislocations and strain fields at a Ge/Si heterostructure interface were investigated experimentally using a combination of high-resolution transmission electron microscopy and quantitative electron micrograph analysis methods. The type of misfit dislocation at the interface was determined to be 60° dislocation and 90° full-edge dislocation. The full-field strains at the Ge/Si heterostructure interface were mapped by using the geometric phase analysis (GPA) and peak pairs analysis (PPA), respectively. The effect of the mask size on the GPA and PPA results was analyzed in detail. For comparison, the theoretical strain fields of the misfit dislocations were also calculated by the Peierls-Nabarro and Foreman dislocation models. The results showed that the optimal mask sizes in GPA and PPA were approximately three tenths and one-tenth of the reciprocal lattice vector, respectively. The Foreman dislocation model with an alterable factor *a* = 4 can best describe the strain field of the misfit dislocation at the Ge/Si heterostructure interface.

## 1. Introduction

Heterostructures have a wide range of applications, including electronic, optoelectronic, and energy conversion devices [1,2,3]. Due to two different lattice parameters, the misfit lattices can induce strain at the heterostructure interface. So far, strain has been an effective way to design or improve the performance for nanoscale materials, including electronic properties arising from band structure modification [4], ferroelectricity [5], thermal conductivity [6], and the photoluminescence [7,8]. But on the other hand, lattice mismatch can also induce dislocations at the heterostructure interface, which will break the crystal symmetry, reshape local band gap structures, and seriously affect the photoelectric properties of the heterostructure. Therefore, analyzing misfit dislocation and strain fields at the heterostructure interface is highly significant for the material performance improvement and extensive application potential. Moreover, there are many dislocation models, such as the elastic theory model and discrete model. The Peierls-Nabarro dislocation model [9,10], one of the most important models, has been discussed by numerous researchers [11,12]. Foreman [13] proposed an improved model based on the Peierls-Nabarro dislocation model. So far, which dislocation model is more appropriate for describing the misfit dislocation at the Ge/Si heterostructure interface is still not clear.

X-ray diffraction or neutron scattering can be employed to measure the average elastic strains within the scattering volume. However these two techniques can be only used to determine the macroscopic average elastic strain, as well as mesoscale intergranular strains [14]. For the Ge/Si heterostructure, the strains are located at the interface region with a scale of a few nanometers, which is difficult to measure by X-ray diffraction or neutron scattering technique. So, nanoscale strain measurement techniques are needed. Recent advance in quantitative electron micrograph offered the possibility of locally determining the elastic strain of materials at the nanoscale using high-resolution transmission electron microscopy (HRTEM). Several quantitative electron micrograph analysis methods described in the literatures are based mainly on two different algorithms: *i.e.*, real space and Fourier space algorithms. The peak finding approach based on real space algorithm was proposed by Bierwolf [15], which was developed and applied to semiconductor nanostructures by Rosenauer [16]. A modified peak finding approach was presented by Galindo and named peak pairs analysis (PPA) [17], which has been applied to In(Ga)As/AlGaAs self-assembled semiconductor quantum dots [18], heterostructured wurtzite InAs/InP nanowires [19], and InAs/GaAs (001) interface [20].The geometric phase analysis (GPA) based on Fourier space algorithm [21] has been applied to various systems, such as dislocations [22,23], nanoparticles [24], and Al/Si nanoclusters [25]. Numerical Moiré can be calculated from the geometric phase of the GPA. The numerical Moiré pattern acts as a lens that magnifies not only the lattice spacing but also the deformation. By selecting a geometric phase image, a desired numerical Moiré image corresponding to a group of special crystal planes can be obtained, thus allowing a detailed analysis of this group of crystal planes [26]. For the both methods above, the selection of mask size is a necessary process, which has very important effect on the calculation results of strain fields. However, how the mask will affect the calculation results of the strain fields and the selection of an appropriate mask size are still not clear. In this paper, we performed quantitative strain analysis for the misfit dislocations at a Ge/Si heterostructure interface. The full-field strains were mapped by GPA and PPA, respectively. The selection of mask size for both methods was discussed in detail. The theoretical strain fields were also mapped by Peierls-Nabarro and Foreman dislocation models.

## 2. Theory

### 2.1. Strain of Edge Dislocation Given by Peierls-Nabarro Model

According to the Peierls-Nabarro dislocation model, the strain of an edge dislocation along the *x* direction can be written as:

(1) $$\varepsilon_{xx}=\frac{b}{\pi }\frac{(1-\nu )y}{4{\left(1-\nu \right)}^{2}+y^{2}}$$

where *x* and *y* are the respective right-angle coordinates centered on the dislocation core position, *b* is Burgers vector, and *ν* is the Poisson’s ratio.

### 2.2. Strain of Edge Dislocation Given by Foreman Model

According to the Foreman dislocation model, the strain of an edge dislocation along the *x* direction is given by:

(2) $$\varepsilon_{xx}=\frac{b(1-\nu )}{\pi }\frac{4{\left(1-\nu \right)}^{2}yx^{2}+(2a^{3}-a^{2})y^{3}}{{\left[4{\left(1-\nu \right)}^{2}x^{2}+a^{2}y^{2}\right]}^{2}}$$

where *x* and *y* are the respective right-angle coordinates centered on the dislocation core position, *b* is Burgers vector, *ν* is Poisson’s ratio, and *a* is an alterable factor which can control the dislocation width. The Foreman model becomes the Peierls-Nabarro model when *a* = 1.

## 3. Experimental Methods

### 3.1. Specimen Preparation

An ultra-high vacuum chemical vapor deposition system equipped with pyrolytic BN effusion cells was used to grow Ge films on Si (001) substrate. The system had a base pressure of 3.0 × 10^−8^ Pa. In situ reflection high-energy electron diffraction (RHEED) was used to monitor the growth of the films. Before growth, the Si substrate was cleaned using the Radio Corporation of America (RCA) method, after which the substrate was degassed in a pre-treatment chamber at 400 °C, and then baked in a growth chamber at 930 °C to deoxidize. The Ge films were grown using a two-step process, in which a 40 nm-thick Ge layer was deposited at 200 °C, and then a 400 nm-thick Ge layer was grown at 500 °C to obtain high-quality Ge thin films. Both the RHEED patterns for the Si substrate and Ge films had well-developed 2 × 1 reconstructions. The overall Ge films were of high quality with atomic surfaces [27].

### 3.2. Electron Microscopy

The TEM samples were prepared for cross-sectional imaging along the [$1\bar{1}0$] direction using a standard technique, which involved mechanical grinding followed by ion milling. An HRTEM experiment was performed on a JEM-2010 transmission electron microscope at 200 kV. Images were recorded on a Gatan 1 k × 1 k slow-scan charge-coupled device camera and then processed using GPA and PPA.

### 3.3. Geometric Phase Analysis

GPA works in Fourier space, and involves the filtering of an image with an asymmetric filter centered on a Bragg spot in the Fourier transform of an HRTEM lattice image and performing an inverse Fourier transform. The phase component of the resulting complex image gives information about local displacements in a direction normal to lattice fringes corresponding to the position of the Bragg spot. The geometric phase *P**_g_***(***r***) of these local Fourier components is directly related to the displacement field component, ***u***(***r***), in the direction of the reciprocal lattice vector ***g***. *P**_g_***(***r***) is given by the following [21]:

(3) $$P_{g}(r)=-2\pi g\cdot u(r)$$

The two-dimensional displacement fields are determined by measuring two phase images, namely, *P_g_*_1_(***r***) and *P_g_*_1_(***r***) as follows:

(4) $$u(r)=-\frac{1}{2\pi }[P_{g1}(r)a_{1}+P_{g2}(r)a_{2}]$$

where ***a***_1_ and ***a***_2_ are the basis vectors of the lattices in real space corresponding to the reciprocal lattices defined by ***g***_1_ and ***g***_2_, respectively. Equation (4) is presented in matrix form as follows:

(5) $$\left(\begin{array}{c} u_{x}\\ u_{y} \end{array}\right)=-\frac{1}{2\pi }\left(\begin{array}{cc} a_{1x} & a_{2x}\\ a_{1y} & a_{2y} \end{array}\right)\left(\begin{array}{c} P_{g1}\\ P_{g2} \end{array}\right)$$

Plane strain is then written as:

(6) $$\{\begin{array}{l} \varepsilon_{xx}=\frac{\partial u_{x}}{\partial x}\\ \varepsilon_{yy}=\frac{\partial u_{y}}{\partial y}\\ \varepsilon_{xy}=\frac{1}{2}\left(\frac{\partial u_{x}}{\partial y}+\frac{\partial u_{y}}{\partial x}\right) \end{array}$$

So far, GPA has been developed as commercial software by HREM Research Inc., which is a plug-in for the image processing package Gatan DigitalMicrograph.

### 3.4. Peak Pairs Analysis

PPA is a real space procedure for strain mapping. PPA works on a Bragg-filtered image, locating pairs of peaks along a predefined direction and distance in the affine transformed space defined by a pair of basis vectors. First, a Wiener filter is used to reduce the noise of the experimental image and detect the local intensity maxima in the filtered image. The second step is to determine two non-collinear basis vectors $\vec{a}=(a_{x},a_{y})$ and $\vec{b}=(b_{x},b_{y})$ that will be used as the references to which determine the strain of the specimen. Once the reference vectors are chosen, these can be used to define an affine transformation. The next step in the procedure is the identification of pairs of peaks using the chosen basis vectors and the intensity maxima set in the image. The identification of pairs of peaks along two non-collinear directions enables us to determine precisely the strain fields. The strain components can be calculated by solving the following set of linear equations [17]:

(7) $$\begin{array}{c} u_{x}=a_{x}\varepsilon_{xx}+a_{y}\varepsilon_{xy}\\ u_{y}=a_{y}\varepsilon_{yy}+a_{x}\varepsilon_{yx}\\ v_{x}=b_{x}\varepsilon_{xx}+b_{y}\varepsilon_{xy}\\ v_{y}=b_{y}\varepsilon_{yy}+b_{x}\varepsilon_{yx} \end{array}\}$$

(8) $$\begin{array}{l} \left[\begin{array}{l} \varepsilon_{xx}\\ \varepsilon_{xy} \end{array}\right]={\left[\begin{array}{cc} a_{x} & a_{y}\\ b_{x} & b_{y} \end{array}\right]}^{-1}\cdot \left[\begin{array}{l} u_{x}\\ v_{x} \end{array}\right]\\ \left[\begin{array}{l} \varepsilon_{yx}\\ \varepsilon_{yy} \end{array}\right]={\left[\begin{array}{cc} a_{x} & a_{y}\\ b_{x} & b_{y} \end{array}\right]}^{-1}\cdot \left[\begin{array}{l} u_{y}\\ v_{y} \end{array}\right] \end{array}$$

where $\left(u_{x},u_{y}\right)$ and $\left(v_{x},v_{y}\right)$ are the coordinates of the displacement with respect to the reference vector $\vec{a}=(a_{x},a_{y})$ and $\vec{b}=(b_{x},b_{y})$, respectively. Once the lattice distortion tensor is determined for each maximum in the image, and by simple interpolation, the continuous distortion fields can be determined [17]. PPA had been developed as a specific software package, called Strain Determination Software, which is available from the website of Galindo [28]. Recently, it also has been developed into commercial software by HREM Research Inc., which is a plug-in for the image processing package Gatan Digital Micrograph.

## 4. Results and Discussion

### 4.1. Type of Misfit Dislocations at the Ge/Si Heterostructure Interface

**Figure 1:**
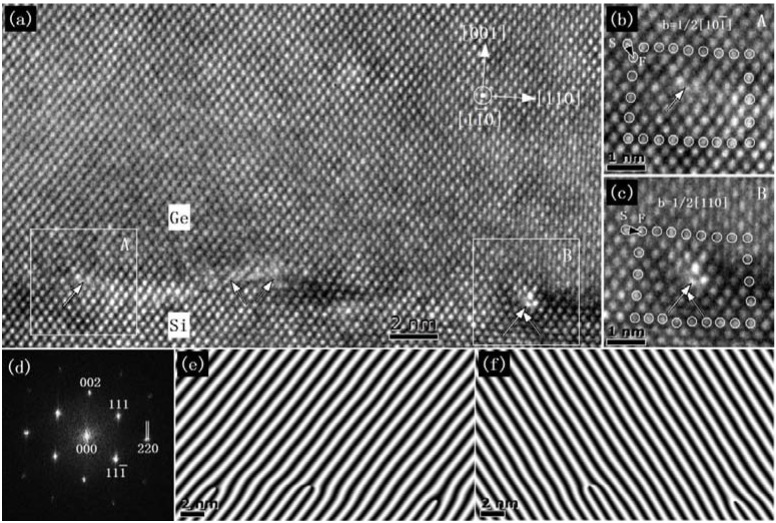
Electron micrograph of a Ge/Si heterostructure: (**a**) high-resolution transmission electron microscopy (HRTEM) image of the Ge/Si heterostructure; (**b**) Burgers circuit around a 60° dislocation; (**c**) Burgers circuit around a 90° full-edge dislocation; (**d**) fast Fourier transform (FFT) pattern of the HRTEM image of the Ge/Si heterostructure; (**e**) 3× numerical moiré pattern of the (**e**) ($11\bar{1}$) crystal plane; (**f**) 3× numerical moiré image of the (111) crystal plane.

**Figure 2:**
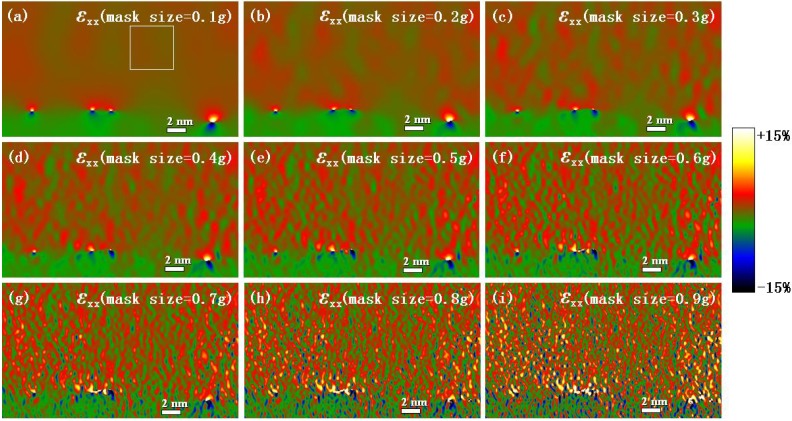
Experimental strain components *ε_xx_* at the Ge/Si heterostructure interface corresponding to the different mask sizes determined by geometric phase analysis (GPA).

Figure 1a shows an HRTEM image of the Ge/Si heterostructure at the [$1\bar{1}0$] zone axis. The upper region is Ge film, and the lower region is Si substrate. It is seen that the only defects in the Ge/Si heterostructure are the misfit dislocations located at the Ge/Si interface. Several misfit dislocation cores marked by white arrows at the Ge/Si interface can be identified clearly. The type of these misfit dislocations can be directly determined from the HRTEM images by drawing a Burgers circuit around the dislocations. Two examples of the misfit dislocations (boxed area A and B in Figure 1a) are enlarged and shown in Figure 1b,c, where the sense vector points into the page and the direction of the Burgers circuit is clockwise, according to the right-hand/finish-start convention [29]. The Burgers vector of the left dislocation can be determined to be 1/2[$10\bar{1}$] (Figure 1b), which is a 60° dislocation [30]. Given that the atomic arrangements of the 60° dislocation were projected onto the HRTEM image, the Burgers vector decided by the current Burgers circuit in Figure 1b is the edge component corresponding to the Burgers vector 1/2[$10\bar{1}$] in the projection plane [31]. The extra half-plane is located at the ($11\bar{1}$) plane, and the Burgers vector lies in the (111) plane. The edge component can be decided to be 1/4[$11\bar{2}$]. The Burgers vector of the right dislocation can be determined to be 1/2[110] (Figure 1c), which is a 90° full-edge dislocation formed by the reaction of two 60° dislocations (marked by white arrows), according to the following dislocation reaction: 1/2[$10\bar{1}$](111) + 1/2[011]($11\bar{1}$) = 1/2[110](001) [32]. Figure 2d shows the fast Fourier transform (FFT) pattern of the HRTEM image (Figure 1a). The diffraction spots in the FFT image were divided into paired separate spots (for example, indicated by two white lines for the paired spots of 220), which correspond to those of the Si and Ge crystals. The distances from the 000 spot to the paired separate spots of 220 in the FFT pattern were calculated. Calculation results show that the difference in the lattice constant between Si and Ge is approximately 4%. This difference is close to the Ge–Si lattice mismatch, indicating that the mismatch strain of the Ge films is almost relaxed [33].

To visualize the atomic arrangement of these dislocations, the numerical moiré images for the two {111} planes were calculated by using a magnification factor of three, as shown in Figure 1e and f. Three 60° dislocations with Burgers vector of 1/2[$10\bar{1}$] can be identified clearly in the ($11\bar{1}$) plane (Figure 1e), and two 60° dislocations with Burgers vector of 1/2[011] can be identified clearly in the (111) plane (Figure 1f). The exact positions of each 60° dislocation can also be decided by the two numerical moiré images.

### 4.2. Effect of Mask Size on the Strain Calculation Results with GPA

Taking the *x*-axis parallel to [110] and the *y*-axis parallel to [001], the full-field strain was first calculated by the GPA method. Considering that the mask size is an important parameter in GPA, Figure 2 shows the strain components *ε_xx_* at the Ge/Si heterostructure interface for different mask sizes to analyze the effect of the mask size on the GPA results. It can be noticed that there are several eight-shaped convergence regions of strain around the misfit dislocation cores. At the upper region (Ge), the strains are positive and tensile, and the lattice is compressive at the lower region (Si). The results also show that with the increase of mask size, the smoothness of the calculation results with GPA worsened.

**Figure 3:**
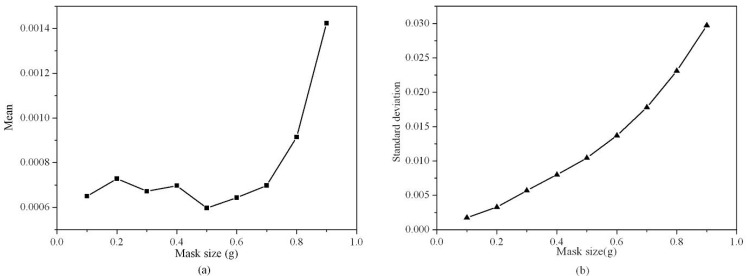
Mean and standard deviation of strain component *ε_xx_* in reference area by using GPA *vs.* mask size: (**a**) Mean *vs*. mask size; (**b**) Standard deviation *vs.* mask size.

To determine the suitable mask size, the means and standard deviations of strain components *ε_xx_* in the reference area (unstrained area, boxed area in Figure 2a) by using GPA for different mask sizes are shown in Figure 3a,b, respectively. It can be noted when the mask size was less than 0.5 *g* (*g* is the module of the reciprocal lattice vector), the mean was small and changed slightly, but when the mask size was larger than 0.5 *g*, the mean increased rapidly with the mask size. Meanwhile, the standard deviation increased steadily with the mask size. When the mask size was 0.4 *g*, the standard deviation had reached 0.0080. From Figure 2, the artifacts in the strain map were very obvious when mask size was more than 0.5 *g*, which is corresponding to the larger standard deviation. These artifacts are due mainly to noise. In HRTEM images, noise is caused by the electron source, the specimen (thickness, surface roughness, amorphous layers, and contamination), and the detector. Increasing mask size will increase noise, so the artifacts in strain map increased. On the other hand, reducing the mask size can reduce the noise and increase precision, but at the expense of spatial resolution [34]. By comprehensive consideration of the mean and standard deviation, the suitable range of the mask size should be less than 0.4 *g*.

### 4.3. Effect of Mask Size on the Strain Calculation Results with PPA

**Figure 4:**
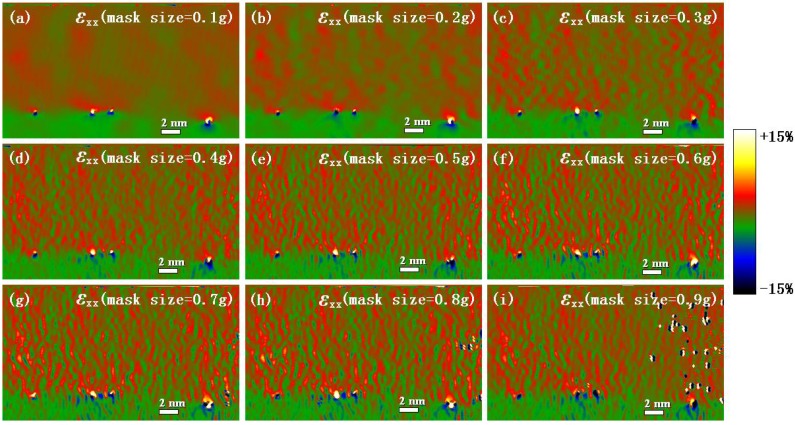
Experimental strain components *ε_xx_* at the Ge/Si heterostructure interface corresponding to different mask sizes determined by peak pairs analysis (PPA).

**Figure 5:**
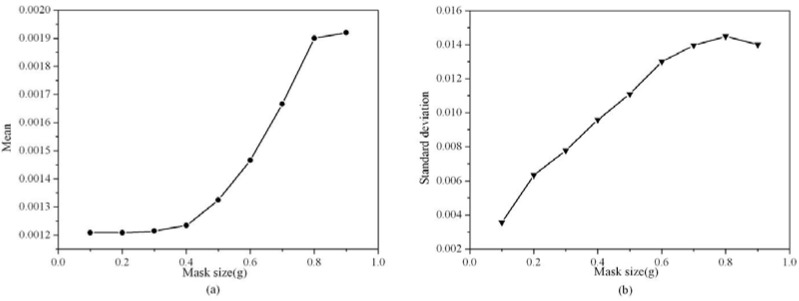
Mean and standard deviation of strain component *ε_xx_* in the reference area by using PPA *vs.* mask size: (**a**) Mean *vs.* mask size; (**b**) Standard deviation *vs.* mask size.

Similar to GPA, the mask size of Bragg filter in PPA also need be chosen properly. The strain components *ε_xx_* at the Ge/Si heterostructure interface for the different mask sizes of Bragg filter are shown in Figure 4. It can be noted that with the increase of mask size, the smoothness of the calculation results with PPA worsened, which is similar to the GPA results. The means and standard deviations of strain components *ε_xx_* in the reference area, which is the same area as that in Figure 2a, for different mask sizes are also shown in Figure 5a,b, respectively. It can be noted that when the mask size of the Bragg filter was less than 0.4 *g*, the mean was small and changed slightly. However, when the mask size was more than 0.4 *g*, the mean increased rapidly with the mask size. Meanwhile, the standard deviation increased steadily with the mask size. When the mask size was 0.3 *g*, the standard deviation has reached 0.0078. From Figure 5, the undesirable artifacts in strain map were very obvious when mask size was more than 0.3 *g*, which is corresponding to the larger standard deviation. The mask size determines the range of frequencies that are removed by the filter. The larger mask size produce noisy images and artifacts were induced. By comprehensive consideration of the mean and standard deviation, the suitable range of the mask size of the Bragg filter should be less than 0.3 *g*.

### 4.4. Theoretical Calculation

**Figure 6:**
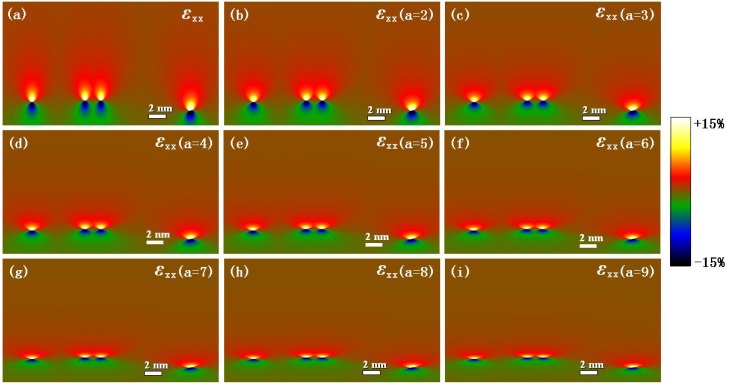
Theoretical strain fields at the Ge/Si heterostructure interface given by the Peierls-Nabarro and Foreman dislocation models: (**a**) Strain component *ε_xx_* given by Peierls-Nabarro model; (**b**–**i**) Strain components *ε_xx_* corresponding to different values of factor *a* given by Foreman model.

To compare the experimental results with the theoretical models, the strain components *ε_xx_* at the Ge/Si heterostructure interface given by the Peierls-Nabarro and Foreman dislocation models are mapped and shown in Figure 6. Figure 6a shows the strain component *ε_xx_* calculated using the Peierls-Nabarro dislocation model. Figure 6b–i demonstrate the strain components *ε_xx_* given by the Foreman model, with corresponding different alterable factors ranging from *a* = 2 to *a* = 9. The strain maps of the misfit dislocations are all eight-shaped. However, the eight-shaped strain maps became wider and shorter and the dislocation width increased when the factor *a* of the Foreman model increased.

To find the appropriate alterable factor *a* of Foreman model, the F-test was employed to analyze the degree of agreement between the experimental results and the theoretical models. In the *F*-test, null hypothesis is defined as $H_{0}:\sigma_{1}^{2}=\sigma_{2}^{2}$, and the alternative hypothesis is defined as $H_{1}:\sigma_{1}^{2}\neq \sigma_{2}^{2}$, where $\sigma_{1}^{2}$ and $\sigma_{2}^{2}$ are the population variances corresponding to the experiment and the theoretical model, respectively [35]. Test statistic *F* is defined as $F=s_{1}^{2}/s_{2}^{2}$, where $s_{1}^{2}$ is the sample variance corresponding to the experiment, and $s_{2}^{2}$ is the sample variance corresponding to the theoretical model. The size of these strain maps is *N* = 718 × 412 = 295,816. Thus, the critical values of the *F* distribution for a two-tailed test are *F*_0.025_(295815,295815) = 1.00723 and *F*_0.975_(295815,295815) = 0.99282 at the 0.05 significance level. Based on the previous analysis on mask size (Section 4.2 and Section 4.3), the *F*-test only compares the experimental results with the suggested GPA and PPA results.

Table 1 shows the calculated *F* values for the GPA results and the Foreman model with different *a* values (*a* = 1 corresponds to the Peierls-Nabarro model). When the mask size is 0.1 *g* and 0.2 *g*, it is found that there is no *F* value can satisfy the inequality $F_{0.975}<F<F_{0.025}$, which suggests that the mask sizes of 0.1 *g* and 0.2 *g* in GPA are not optimal. When the mask size is 0.3 *g*, it is found that there is only one value *F*(0.3*g*)_*a*=4_ that can satisfy the inequality $F_{0.975}<F<F_{0.025}$, which suggests that the mask size of 0.3 *g* in GPA is the optimal mask size, and the Foreman dislocation with *a* = 4 is appropriate for the misfit dislocation. Similarly, Table 2 shows the calculated *F* values for the PPA results, and the Foreman model with different *a* values (*a* = 1 corresponds to the Peierls-Nabarro model). When the mask size is 0.1 *g*, it is found that there is only one value *F*(0.1*g*)_*a*=4_ that can satisfy the inequality $F_{0.975}<F<F_{0.025}$, which suggests that the mask size of 0.1 *g* in PPA is the optimal mask size, and the Foreman dislocation with *a* = 4 is appropriate for the misfit dislocation. When the mask size is 0.2 *g*, it is found that there is no *F* value can satisfy the inequality $F_{0.975}<F<F_{0.025}$, which suggests that the mask size of 0.2 *g* in PPA is not an optimal mask size. So, the *F*-test indicates that the variances in the GPA calculation results corresponding to the mask size of 0.3 *g*, in the PPA results corresponding to the mask size of 0.1 *g*, and in the theoretical results by the Foreman model with *a* = 4, have no significant difference at the 0.05 significance level. Therefore, the optimal mask sizes in GPA and PPA are approximately 0.3 *g* and 0.1 *g*, respectively. The Foreman model with *a* = 4 can best describe the strain fields of the misfit dislocations at the Ge/Si heterostructure interface.

**Table 1:** Calculated *F* values for the GPA results and the Foreman model with different *a* values.

*a*	1	2	3	4	5	6	7	8	9
*F*(0.1*g*)	0.70402	0.76388	0.82277	0.86558	0.89697	0.92071	0.93912	0.95377	0.96563
*F*(0.2*g*)	0.72084	0.78213	0.84243	0.88626	0.91840	0.94270	0.96155	0.97655	0.98870
*F*(0.3*g*)	0.80963	0.87846	0.94619	0.99542	1.03152	1.05881	1.07999	1.09683	1.11048

**Table 2:** Calculated *F* values for the PPA results and the Foreman model with different *a* values.

*a*	1	2	3	4	5	6	7	8	9
*F*(0.1*g*)	0.81862	0.88822	0.95670	1.00648	1.04298	1.07058	1.09199	1.10902	1.12282
*F*(0.2*g*)	1.01457	1.10084	1.18571	1.24740	1.29264	1.32684	1.35337	1.37449	1.39158

## 5. Conclusions

The full-field strain at a Ge/Si heterostructure interface was quantitatively analyzed. The type of misfit dislocations at the Ge/Si heterostructure interface was directly identified from the HRTEM image. The full-field strain was mapped by GPA and PPA, respectively. The effect of mask size on the GPA and PPA results was analyzed in detail. For comparison, the theoretical strain fields at the Ge/Si heterostructure interface were given by the Peierls-Nabarro and Foreman dislocation models. For both GPA and PPA, the smoothness of the calculation results worsened with the increase of mask size. The optimal mask sizes in GPA and PPA are approximately 0.3 *g* and 0.1 *g*, respectively. The Foreman model with alterable factor *a* = 4 can best describe the strain fields of the misfit dislocations at the Ge/Si heterostructure interface.
